# Investigating PM_2.5_ toxicity in highly polluted urban and industrial areas in the Middle East: human health risk assessment and spatial distribution 

**DOI:** 10.1038/s41598-023-45052-z

**Published:** 2023-10-19

**Authors:** Babak Goodarzi, Maryam Azimi Mohammadabadi, Ahmad Jonidi Jafari, Mitra Gholami, Majid Kermani, Mohammad-Ali Assarehzadegan, Abbas Shahsavani

**Affiliations:** 1 Research Center for Environmental Health Technology, Iran University of Medical Sciences, Tehran, Iran; 2 Department of Environmental Health Engineering, School of Public Health, Iran University of Medical Sciences, Tehran, Iran; 3 Department of Environmental Health Engineering, School of Public Health, Hormozgan University of Medical Sciences, Bandar Abbas, Hormozgan Iran; 4 Immunology Research Center, Institute of Immunology and Infectious Diseases, Iran University of Medical Sciences (IUMS), Tehran, Iran; 5 Air Pollution Research Center, Iran University of Medical Sciences, Tehran, Iran; 6 Environmental and Occupational Hazards Control Research Center, Shahid Beheshti University of Medical Sciences, Tehran, Iran

**Keywords:** Environmental sciences, Natural hazards, Biomarkers, Molecular medicine

## Abstract

Exposure to particulate matter (PM) can be considered as a factor affecting human health. The aim of this study was to investigate the concentration of PM_2.5_ and heavy metals and their influence on survival of A549 human lung cells in exposure to PM_2.5_ breathing air of Ahvaz city. In order to assess the levels of PM_2.5_ and heavy metals, air samples were collected from 14 sampling stations positioned across Ahvaz city during both winter and summer seasons. The concentration of heavy metals was determined using ICP OES. Next, the MTT assay [3-(4,5-dimethylthiazol-2-yl)-2,5-diphenyltetrazolium bromide] was employed to ascertain the survival rate of A549 cells. The findings from this research demonstrated that average PM_2.5_ of the study period was (149.5 μg/m^3^). Also, the average concentration of PM_2.5_ in the urban area in winter and summer was (153.3- and 106.9 μg/m^3^) and in the industrial area this parameter was (191.6 and 158.3 μg/m^3^). The average concentration of metals (ng/m^3^) of urban areas against industrial, Al (493 vs. 485), Fe (536 vs. 612), Cu (198 vs. 212), Ni (128 vs. 129), Cr (48.5 vs. 54), Cd (118 vs. 124), Mn (120 vs. 119), As (51 vs. 67), Hg (37 vs. 50), Zn (302 vs. 332) and Pb (266 vs. 351) were obtained. The results of the MTT assay showed that the highest percentage of cell survival according to the exposure concentration was 25 > 50 > 100 > 200. Also, the lowest percentage of survival (58.8%) was observed in the winter season and in industrial areas with a concentration of 200 μg/ml. The carcinogenic risk assessment of heavy metals indicated that except for Cr, whose carcinogenicity was 1.32E−03, other metals were in the safe range (10^–4^–10^–6^) for human health. The high concentration of PM_2.5_ and heavy metals can increase respiratory and cardiovascular diseases and reduce the public health level of Ahvaz citizens.

## Introduction

PM_2.5_, which refers to fine particulate matter with an aerodynamic diameter less than 2.5 μm, is recognized as a prominent environmental hazard^1–5^; resulting in approximately 4.2 million deaths in 2019, globally^2,6^. There is a correlation between PM_2.5_ and elevated occurrences of respiratory illnesses such as asthma, bronchitis, chronic obstructive pulmonary disease (COPD), and lung cancer^7–9^, cardiovascular diseases and mortality have been proven over the recent years^4,10,11^. Of particular interest is the fact that 99% of the global population inhales PM_2.5_ levels that surpass the Air Quality Guideline set by the World Health Organization (5 µg m^−3^)^1,12^. Although PM_2.5_ constitutes a small percentage of suspended particles^3^, large surface area^13,14^, absorption of various chemicals^2,14^, the ability to penetrate into the lower parts of the lungs^8,15–18^ and disrupt the gas exchange makes PM_2.5_ to cause major health and respiratory-related disorders^8,13,18^. PM_2.5_ enter the atmosphere following natural and man-made activities^2,19,20^. According to the source, location, production time and atmospheric conditions, PM_2.5_ have different chemical structure with different health effects^10,15,19,21^. Heavy metals (HMs) and polycyclic aromatic hydrocarbons (PAHs) are amongst the most important chemical components bounded to PM_2.5_^3,15,22^, which has the ability to deeply penetrate into the lungs and bloodstream^21,23^. These compounds can lead to DNA damage, cell death and genotoxicity^15,16^. HMs accumulate in various fat tissues; these compounds difficult to break down have a serious impact on human health, causing cancer after reaching a certain dose^23^. A549 pulmonary epithelial cell line plays an vital role in protecting other body cells due to exposure to particles and surfactant secretion, stimulation of the immune system and identification the pathogens and warning to leukocyte cells^24–26^. A549 cells are extensively utilized in toxicological research to investigate the impact of particles on the human body within an in-vitro setting^4,27^.

MTT assay is a colorimetric method used to survey the cell survival and cytotoxic effects of chemical compounds influencing on cells following exposure to particles^28,29^. Although a growing number of studies have used this approach/method to investigate the effects of PM_2.5_ on cell survival, results have been mixed around the world and thus the overall evidence remains inconclusive. For instance, Perrone et al.^30^ conducted a study to examine the impact of PM1 and PM_2.5_ present in the ambient air of Milan during both winter and summer seasons on the A549 cell line. The authors reported that cell survival percentage in the Winter was higher than those in summer. Furthermore, Chen et al.^31^ conducted a study to assess the toxicity of PM_2.5_ present in the ambient air of Nanjing, China during winter and summer. Their findings revealed that PM_2.5_ particles during the winter season exhibited a higher level of toxicity compared to those observed during the summer season.

Within this framework, the present study represents a pioneering effort aimed at conducting a comprehensive examination of the impacts of ambient PM_2.5_ in Ahvaz, which is recognized as one of the most heavily polluted cities^32^.

To the best of our knowledge, this study, for the first time investigate the concentration, spatial distribution, seasonal changes of PM_2.5_ and heavy metals, the carcinogenic and non-carcinogenic risk of heavy metals bounded PM_2.5_ and the survival rate of A549 human lung cells contacted to the PM_2.5_ in all areas of Ahvaz city as the mega city considering land use (urban and industrial). Furthermore, the additional aims of present research were: (1) to assess the concentration and spatial distribution of PM_2.5_ and heavy metals associated with PM_2.5_ during both the summer and winter seasons; (2) to conduct a human risk assessment of exposure to heavy metals, and 3) to analyze the toxicity of PM_2.5_ using the A549 human lung cell line.

## Materials and methods

### Study area and sampling station

**Figure 1:**
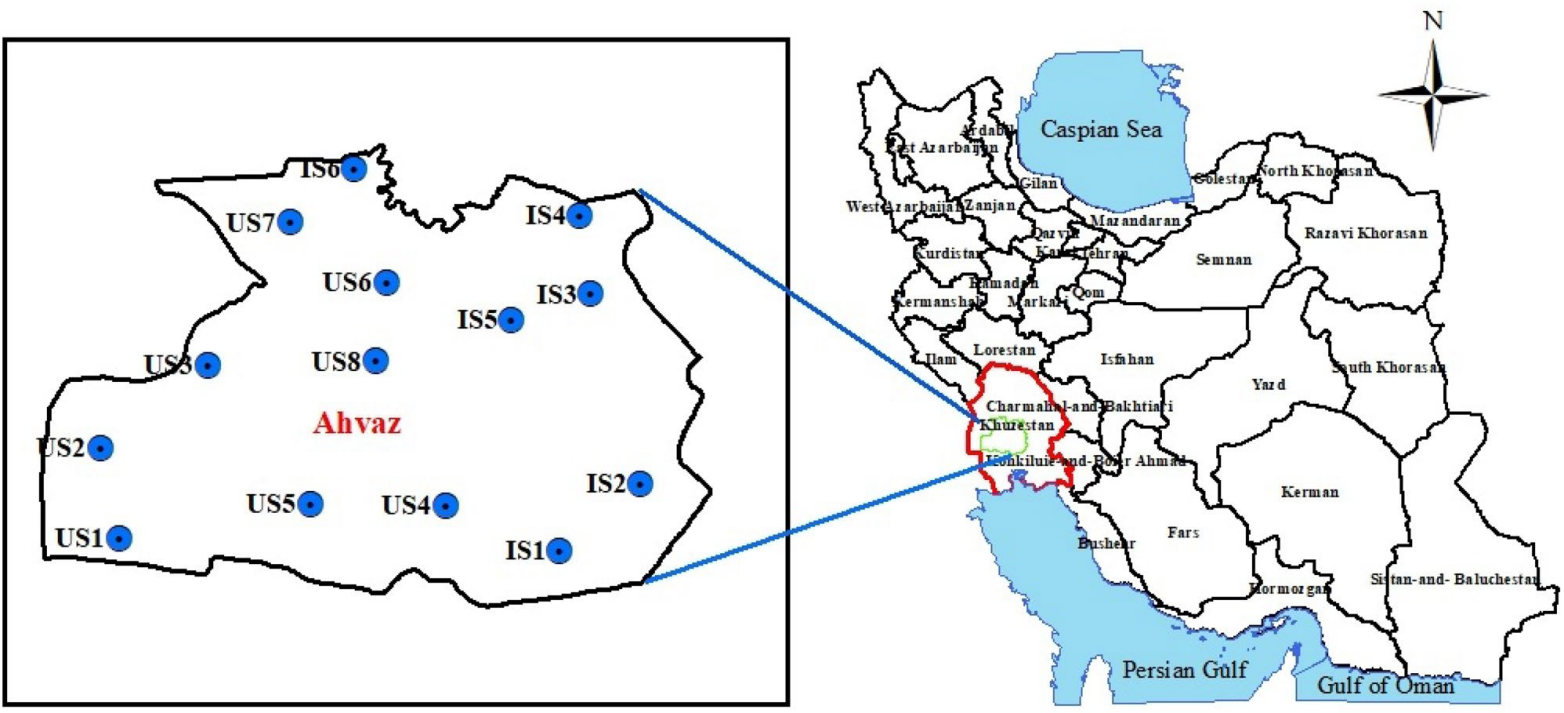
The study area and sampling stations.

The sampling sites were selected in Ahvaz city, located at 31.3183° N, 48.6706° E longitude and latitude, respectively with elevation of 12 m above the sea level in southwest of Iran. Ahvaz, with a population of around 1.5 million, is recognized as one of the seven major cities in Iran. Based on meteorological records, Ahvaz experiences its coldest months, with an average temperature of 12.4 °C, in January and February, while July and August are the hottest months with an average temperature of 38.6 °C. The average annual rainfall is reported to be 213 mm. Ahvaz city is widely recognized as one of the highly polluted urban areas in both Iran and the world, primarily attributed to factors such as heavy traffic, extensive reliance on fossil fuels, and the presence of numerous industrial activities, including power plants, steel, asphalt, carbon block and pipe manufacturing factories, as well as numerous petrochemical industries and refineries. According to World Health Organization (WHO), Ahvaz city was the most polluted city in the world in terms of ambient PM_10_ in 2011^32^. In this study, 14 stations including 8 urban stations (US1 to US8) and 6 industrial stations (IS1 to IS6) were sampled in two summer and winter seasons. The stations were chosen to be evenly distributed throughout the city. The sampling was done at a height of 3 m from the ground. Of note, the location of sampling station for urban (stations far away from main streets with heavy traffic and near residential areas and industrial area) and industrial area (stations located in the vicinity of a source or industrial area) were selected according to guidelines^33^. Figure 1 shows the study area and sampling stations.

### Sampling procedure

During the winter of 2020 and summer of 2021, this research was undertaken in Ahvaz, focusing on two distinct areas: industrial sites comprising of six stations and urban sites consisting of eight stations. A total of 28 samples were gathered across the two seasons, encompassing both winter and summer. Sampling of PM_2.5_ followed the guidelines outlined in EPA TO/13A, employing a peripheral pump (Leland Legacy (SKC)) coupled with a personal modular impactor (PMI_2.5_) set at a flow rate of 5 L/min. This sampling process spanned a duration of 24 h and utilized a 37 mm diameter fiberglass filter with a pore size of 1 µm. Throughout the sampling period, temperature and pressure measurements were documented using a Lutron model PHB 318 thermometer. Additionally, relative humidity, precipitation, cloud cover, and wind speed data were obtained from the Iran Meteorological Organization (IMO). It is important to note that before and after the sampling, all devices and equipment used were calibrated. To determine the PM_2.5_ concentrations, fiberglass filters were weighted before and after sampling using a RADWAG digital scale (AS 220.R2) with an accuracy of 0.0001 and placed in a desiccator (temperature 22–24 °C and relative humidity of 35–45%) for 24 h. The flow rate of the pump was measured using a rotameter both at the start and conclusion of the sampling process, allowing for the calculation of the average flow rate. Subsequently, the PM_2.5_ concentration was determined by subtracting the secondary weight from the primary weight of the filters, employing the formula provided below^34^.

$${\mathrm{C}}_{\mathrm{PM}2.5}=\frac{(\mathrm{Wf}-\mathrm{Wi})\times {10}^{6}}{\mathrm{V}}$$

where C_PM2.5_ is the concentration of PM_2.5_ (µg/m^3^), Wf and Wi are the filter weight at the end and start of sampling (gr), and V denotes the sampling air volume (m^3^).

### PM_2.5_ extraction

To obtain particles for in vitro exposure, filters collected from various land uses were transferred into 15-mL Falcon tubes. Subsequently, 5 mL of deionized water was added to the Falcon tubes, which were then subjected to a 30-min ultrasonic bath. This process was repeated three times^35^. At this stage, the filters were separated from the Falcon tubes and the suspension of particles was transferred to the freeze dryer under vacuum conditions in order to remove the water for 24 h at − 40 °C. Then, the prepared samples were stored at − 20 °C until cell exposure experiments. In this study, according to the season (summer and winter) and the land uses (urban and industrial), four types of PM_2.5_ extractions were obtained.

### Cell culture, PM_2.5_ exposure and cell survival percentage

For the purpose of conducting cell culture experiments in this study, the A549 human lung epithelial cell line was obtained from the National Cell Bank of Iran (NCBI) at the Pasteur Institute. A549 cells were cultivated in a Dulbecco's modified Eagle's medium (DMEM) (Ana Cell, Iran) culture medium supplemented with 1% penicillin/streptomycin, and 10% fetal bovine serum (FBS). The cell culture was maintained in an incubator set at 37 °C, 95% humidity, and 5% CO_2_. MTT (Sigma-Aldrich, USA) colorimetric assay was used to determine cell survival. The MTT reagent was prepared at 5 mg/mL in phosphate buffered saline (PBS). After several times of cell culture and reaching exponential growth conditions and when 80% of the flask space was filled (80% confluency), A549 cells were trypsinized and counted with Neobar slides. Then, 100 μl cell suspension with a concentration of 2 × 10^4^ was cultured in each well of 96- well flat-bottomed plates. After 24 h (sticking the cells to the bottom of the plate), the cells of each well were exposed to 100 µL of four types of PM_2.5_ extraction in different concentrations (25, 50, 100 and 200 µg/ml) for 24 and 48 h. The experiment was conducted in triplicate under varying conditions. Following exposure periods of 24 and 48 h, the cells were rinsed with Phosphate-buffered saline (PBS). Subsequently, 10 μl of MTT and 90 μl of culture medium were introduced into each well, and the plate was incubated at 37 °C with 5% CO2 for 4 h. The supernatant was then discarded, and 100 μl of dimethyl sulfoxide (DMSO) was added to the wells. All samples were performed in triplicate. The plate was then gently shaken at 250 rpm at 4 °C for 30 min in a dark setting until the crystals were fully dissolved. The absorbance of each well was assessed using an ELISA device (BIOHIT PLC, Helsinki, Finland) at a wavelength of 570 nm. The obtained data were then compared to the control group, which represented 100% viability, and the results were reported according to the following formula^35,36^.

$$\mathrm{Cell viability\% }=\frac{(\mathrm{Io}-\mathrm{I})\times 100}{\mathrm{Io}}$$

where I = Absorbance of tetrazolium salt solution in DMSO containing cell exposed with PM sample, I0 = Absorbance of tetrazolium salt solution in DMSO containing cells without PM sample.

### Heavy metals measurement

The concentration of 11 heavy metals bounded with PM_2.5_ (Cu, Zn, Hg, As, Mn, Cd, Cr, Ni, Cu, Fe and Al) was determined using an ICP OES (Optima 8000, PerkinElmer model, USA). The ideal operational parameters were as follows: generator power set at 1.5 kW, generator frequency at 40 MHz, plasma gas flow rate of 8 L per minute, and pump speed adjusted to 1 ml per minute. To quantify the concentration of heavy metals associated with PM_2.5_, half of the filters were fragmented into small segments and deposited into a Teflon container. Then, the combination of 3 mL HNO_3_ (65–68%) and 1 ml hydrochloride (36–38%) was added to the filters and were digested for 2 h in an ultrasonic water bath at a temperature of 90 °C^37,38^. In order to remove acids, the digested solution was dried at 90 to 100 °C under a hood. Then, HNO_3_ and ultrapure water were added in a ratio of 1:9 to the samples and shaken for 15 min. The solution was passed through a syringe head filter featuring a pore size of 2.5 µm for filtration. Subsequently, the samples were diluted with ultrapure water to achieve a final volume of 25 ml and stored at a temperature of 4 °C until injection^39^. Calibration curves were graphed using 6 dots with valid high purity standards purchased from Merck, Germany before injecting main samples. Also, at the same time, a blank fiberglass filter was prepared as a control sample and the concentration of heavy metal was measured for this filter. In order to control the validity of the test, the method of increasing the standard and calculating its recovery percentage was used. Also, by repeating the test for some stations (triplicate) the accuracy of the tests was ensured. In addition, after injecting a number of samples (usually 10 samples), one sample with a certain concentration was injected, if it was within the acceptable range, the rest of the samples were injected.

### Human risk assessment

Human risk analysis is a methodical approach that involves evaluating, managing, and establishing the correlation between environmental pollutants and their impact on human health^40^. In this study, the risk assessment model for human health, as provided by the United States Environmental Protection Agency (EPA)^41,42^, was employed to evaluate the potential health effects, both carcinogenic and non-carcinogenic, resulting from exposure to heavy metals present in the ambient air of Ahvaz city. For this aim, firstly exposure concentration (EC) were calculated using the following equation:

$$EC=\frac{C\times ET\times EF\times ED}{AT}$$

where C indicates pollutant concentration (µg/m^3^), ET is exposure time (h/day), EF is exposure frequency (days/ year), ED represents the duration of exposure (years) which is 2 years for infants (0–2 years), 14 years for children (2–15 years), and 55 years for adults (16–70 years). CF is conversion factor (mg/μg), and AT is averaging time (h).

For carcinogenic effects, lifetime cancer risk (LTCR) was calculated as the following Equation^43,44^:

$$LTCR=EC\times UR$$

where UR is unit risk (m^3^/μg).

Non-cancer risk is quantified by a hazard quotient (HQ) which is the ratio between EC and the inhalation reference concentration (RfC, mg/m^3^) as shown in the following equation:

$$HQ=\frac{EC}{RfC}$$

When the value of HQ exceeds 1, it indicates the presence of potential risks to human health. Conversely, if HQ is equal to or less than 1, it signifies that no potential health effects are anticipated^45,46^. Table. 1 presents the parameters to calculate the carcinogenic and non-carcinogenic risk of heavy metals.

**Table 1:** Parameters to assess the carcinogenic and non-carcinogenic risk of heavy metals.

Variable	Unit	Baby (≥ 2 y)	Child (2–15 y)	Adult(16–70 y)	References
ET (exposure time)	h	8	8	8	^46^
EF (exposure frequency)	day	365	365	365	^47^
ED	year	2	14	54	^47^
AT (non-cancer risk)	h	ED × 365 × 24			^46,47^
AT (cancer risk)	h	70 × 365 × 24			
RfC (mg/m^3^)	Al	5.00E−03			^46–48^
	Ni	1.40E−05			
	Cr	1.00E−04			
	Cd	1.00E−05			
	Mn	5.00E−05			
	As	1.50E−05			
	Hg	3.00E−04			
UR(1/(mg/m3)	Ni	2.40E−04			
	Cr	8.40E−02			
	Cd	1.80E−03			
	As	3.00E−04			

### Spatial distribution of heavy metals

During the study period, GIS software and the inverse distance weight (IDW) technique were employed to ascertain the spatial dispersion of heavy metals associated with PM_2.5_ within Ahvaz city^43,50^.

### Statistical analysis

To examine the variation in cell survival rates among different concentrations, seasons, and areas with distinct uses, a one-way ANOVA test was conducted. Statistical significance was determined using a threshold of *P* < 0.05, indicating significance, while a more stringent threshold of *P* < 0.01 denoted high significance.

### Ethical approval

The study was approved by ethic committee of Iran University of Medical Sciences with ethical code of IR.IUMS.REC.1400.486. 

## Results and discussion

### PM_2.5_ concentration

In both winter and summer seasons, the levels of PM_2.5_ were measured in Ahvaz city, considering both urban and industrial land uses. This measurement was carried out utilizing a peripheral pump known as Leland Legacy (SKC). Table 2 summarizes the PM_2.5_ concentration in different sampling point throughout the Ahvaz city in two seasons (winter and summer). As depicted in Table 2, the mean PM_2.5_ concentration during the winter season was recorded as 153.34 μg/m^3^ in urban areas and 191.65 μg/m^3^ in industrial areas. On the other hand, the average PM_2.5_ concentration during the summer season in these respective areas was 106.97 μg/m^3^ and 158.82 μg/m^3^. In addition, the findings revealed that the average PM_2.5_ concentration in the industrial region exceeded that of the urban area. Overall, the order of average PM _2.5_ concentration was as follows: urban summer < urban winter < industrial summer < industrial winter. These results indicated that in the winter season, due to the decrease in temperature, the increase in the use of fossil fuels, the burning of biomass, the decrease in wind speed, and the creation of inversion conditions, the concentration of particles is higher than that in the summer season^51^. In addition, in winter, the use of fuels such as diesel and coal by industries can be one of the reasons for high particles in these areas^51–53^. Also, according to the Table 2, it can be seen that the highest average concentration was 273.17 µg/m^3^ belonging to station 1 (IS1) in industrial landuse, which is close to the carbon block factory and high traffic. However, the lowest average concentration of PM_2.5_ was 46.29 µg/m^3^ in station 5 (US5), which is located in urban area. Furthermore, the results of this study showed that all 14 monitored stations had concentrations higher than the World Health Organization's Air Quality Guideline (5 µg/m^3^). While there was a distinction in the PM_2.5_ concentration between the summer and winter seasons, no noteworthy variances were detected between the PM_2.5_ levels in urban and industrial areas. Our results are consistent with the previously conducted studies. In a study conducted by Jahid et al. (2021) in Mahshahr, Iran, it was demonstrated that the concentration of PM_2.5_ was elevated during the winter season and at the industrial station compared to other seasons^52^. They reported that Petrochemical industries played an important role in the high concentration of particles. In a study conducted by Kermani et al. in Pakdasht and Varamin, Iran, it was revealed that the concentration of PM_2.5_ during the winter season (68.39 μg/m^3^) was higher compared to the summer seasons. This increase in PM_2.5_ levels was attributed to the utilization of fossil fuels, lower temperatures, and the occurrence of inversions in the region^54^. Also, Rezaei Rahimi et al. study in 2022 in a number of vehicle parking lots in Qom city showed that the concentration of PM_2.5_, PM_10_ suspended particles was higher than the guidelines of the World Health Organization. And these concentrations were higher in the fall season (due to the cold weather) than in the summer season^55^.

**Table 2:** The concentration of PM_2.5_ (μg/m^3^) in both winter and summer seasons in urban and industrial areas.

*Stations*	Winter		Summer	
	Urban	Industrial	Urban	Industrial
*US1*	306.0	–	95.8	–
*US2*	115.7	–	89.6	–
*US3*	141.2	–	99.2	–
*US4*	217.0	–	198.4	–
*US5*	46.3	–	46.2	–
*US6*	68.3	–	46.3	–
*US7*	49.6	–	70.6	–
*US8*	282.5	–	209.6	–
IS1	–	263.4	–	282.9
*IS2*	–	99.2	–	73.1
*IS3*	–	138.9	–	69.4
*IS4*	–	303.0	–	124.0
*IS5*	–	117.7	–	97.5
IS6	–	227.7	–	306.0
Mean	153.3	191.6	106.9	158.8
Total mean	149.5			

### Effect of PM_2.5_ on A549 cells and cell viability

**Figure 2:**
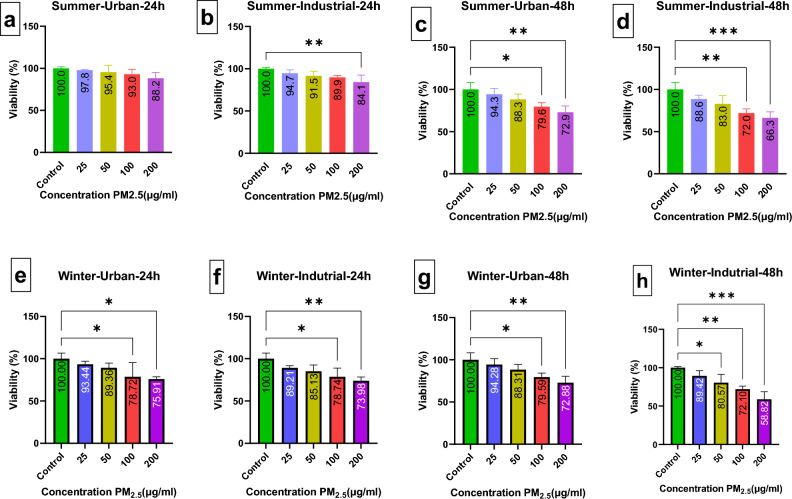
(**a**–**h**) shows different exposure conditions of PM_2.5_ of Ahvaz city on A549 cell line. *P-value < 0.05, **P-value < 0.01 and *** value < 0.001.

In the present study, the effects of PM_2.5_ measured in ambient air of Ahvaz in terms of landuses (Urban and Industrial) on cell survival was in two sampling seasons (hot and cold). In order to achieve this objective, the viability rate of A549 cells was assessed and compared with the control group following exposure to various concentrations of PM_2.5_ (25, 50, 100, and 200 μg/ml) for durations of 24 and 48 h. Figure 2. shows the cell survival following the exposure to different PM_2.5_ concentration in terms of landuses in two seasons. As can be seen in the Fig. 2, the survival percentage of A549 cells experienced a decreasing trend with increasing the PM_2.5_ from 25 to 200 µg/ml. According to Fig. 2(a–d), the highest cell survival in the summer season (%97.8) is related to the concentration of 25 μg/ml with an exposure time of 24 h in the urban area (a) and the lowest cell survival (%66.3) belonged to concentration of 200 μg/ml with an exposure time of 48 in the industrial area (d). According to one-way ANOVA statistical analysis, there was no significant differences between cell survival percentage when exposure to the concentration of 25 μg/ml and control group (*P* value > 0.05). In addition, in the winter season (See Fig. 2E–H), the highest cell survival (%94.3) belonged to concentration 25 μg/ml with exposure time of 24 h in urban area (e) and the lowest survival (% 58.8) was related to concentration 200 with exposure time of 48 h in industrial area (h). Based on our results, it can be stated that ambient PM had been more toxic in winter and industrial areas compared to those in summer and urban areas. The most important reason for the further reduction of cell survival in the winter can be related to the use of fuels such as diesel and coal in the winter, which can produce chemical compounds with higher toxicity^52^. Multiple prior studies have suggested that as the concentration of PM_2.5_ rises and the duration of exposure increases, the rate of cell survival tends to decline^56–59^. Moreover, previously conducted studies around the world showed that ambient PM in summer, with the same concentration and usage, have a lower toxic effects on A549 compared to PM_2.5_ in winter season^20,29,31,60^.

### Heavy metals

**Figure 3:**
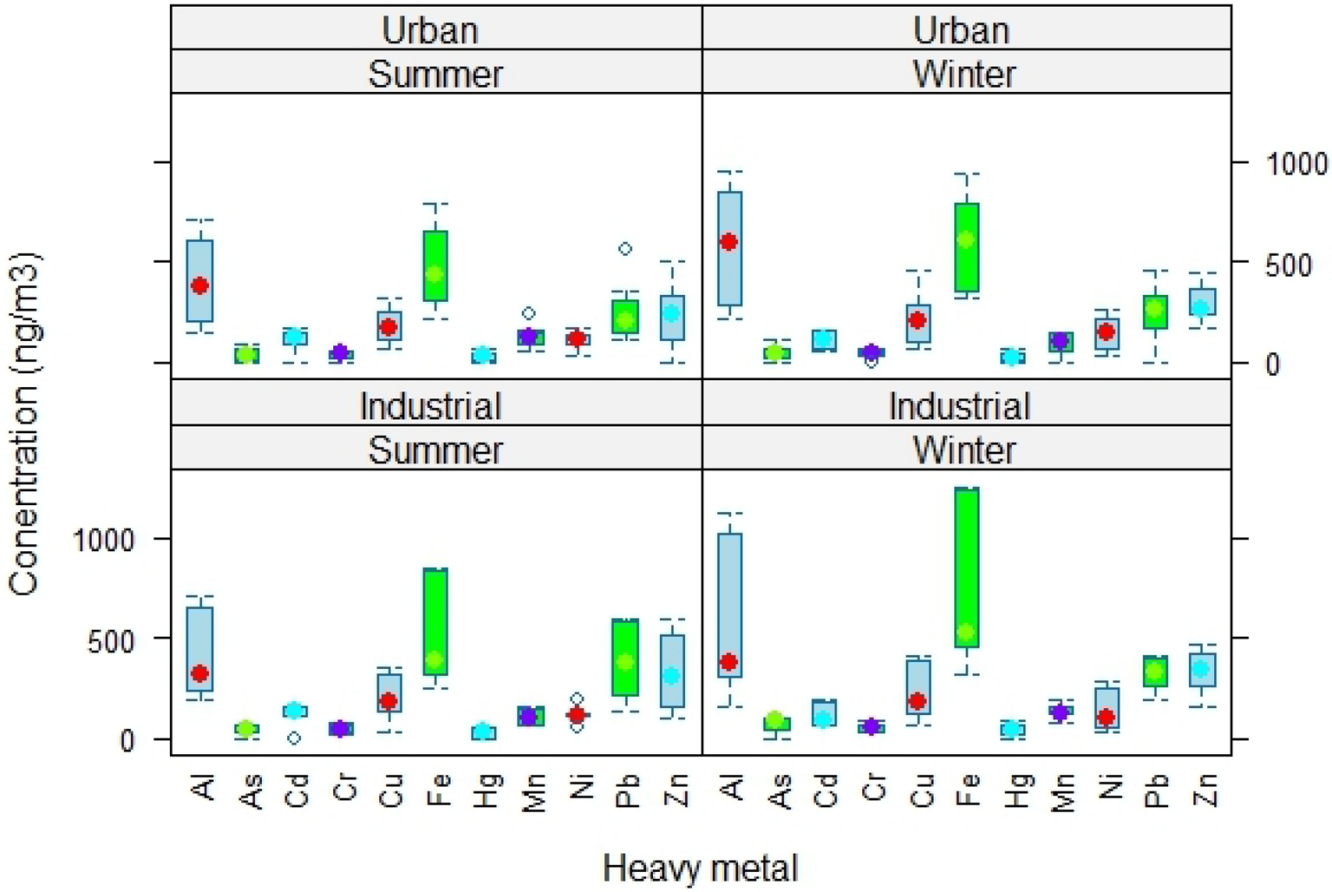
Concentration of heavy metals (ng/m^3^) in winter and summer in urban and industrial areas.

In this study, elev shownen heavy metals including (Al, Fe, Cu, Ni, Cr, Cd, Mn, As, Zn, Hg and Pb) were evaluated (Fig. 3). As shown in Fig. 3, the highest average concentration belonged to Fe (721.7 ng/m^3^) in winter season and industrial area, w shownhile the lowest average concentration belonged to Hg (37.19 ng/m^3^) in summer season and urban area. In addition, the highest average total concentration of all metals belonged to winter season and the industrial area (2742.19 ng/m^3^). Nonetheless, the summer season and urban area exhibited the lowest mean total concentration of all metals (2130.17 ng/m^3^). On the other hand, the order of average concentration of heavy metals from the highest to the lowest were as follows: Fe (568.69 ng/m3), Al (462.595 ng/m3), Zn (316.545 ng/m3), Pb (303.925 ng/m3), Cu (203.41 ng/m3), Ni (128.675 ng/m3), Cd (121.45 ng/m3), Mn (119.665 ng/m3), As (58.47 ng/m3), Cr (50.79 ng/m3), and Hg (44.15 ng/m3). The results indicated that Fe, Al and Zn had the highest concentrations due to their presence in the earth's crust^61^. In the city of Ahvaz, the presence of heavy metals can be due to the high concentration of suspended particles of internal and external origin, as well as the suspension of soil and dust caused by vehicles on the roads^51,62^. Conversely, the presence of industrial facilities such as steel plants, power stations, refineries, and petrochemical plants significantly contributes to the escalation of heavy metal concentrations within this city^63^.The high concentration of Pb in this study can be caused by different industries as well as the low quality of fuel consumed by cars^51^. The higher concentration of heavy metals in the winter season can be due to the phenomenon of inversion and more consumption of fossil fuels by vehicles and factories. According to the findings of Heydari Farsani et al., the concentration of heavy metals in Ahvaz was found to be greater during the winter season compared to the autumn season. The most possible reason behind this are attributed to decrease in temperature, more fuel consumption by vehicles and industries, and the phenomenon of inversion. And the highest concentration of Al metal was expressed as 34.32 ng/m^3^^64^. Due to reasons such as high traffic, the establishment of many industries (especially oil and petrochemical) and the proximity to the deserts of Iraq and Arabia, the concentration of heavy metals in Ahvaz city was higher than the study (Ahmed et al.) in Behbahan^65^.

### Spatial distribution

**Figure 4:**
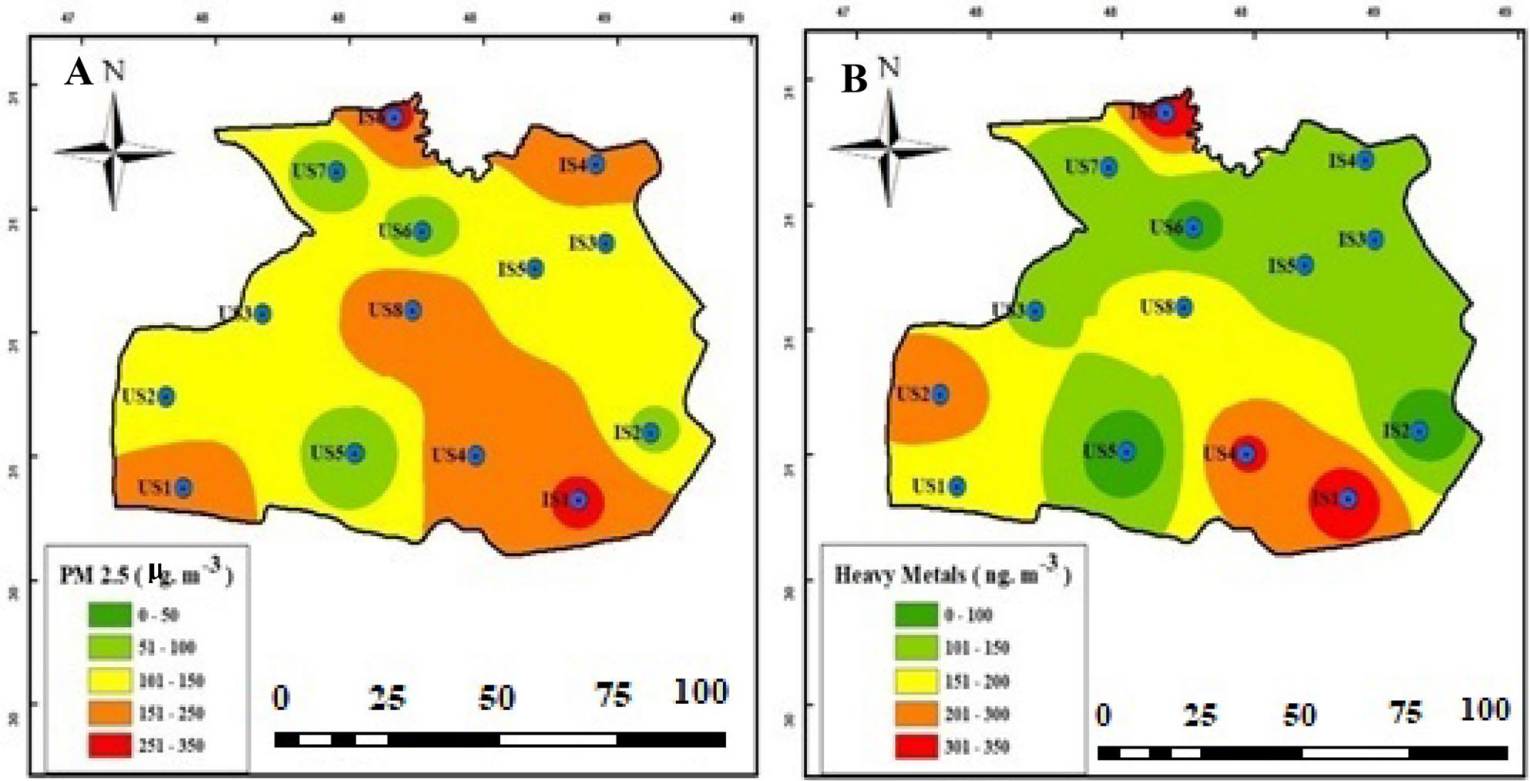
The spatial distribution of PM_2.5_ and HM-bounded in Ahvaz city. (**A**) PM_2.5_ and (**B**) heavy metals.

In this study, GIS software was used to interpolate the measured pollutants to the entire area of ​​Ahvaz city. As can be seen in Fig. 4, stations IS1 and IS6, which are located in industrial areas, had the highest average concentrations of PM_2.5_ (273.2 and 266.9 μg/m^3^) and heavy metals (359.9 and 373.2 ng/m^3^). This shows that the citizens near the stations IS1 and IS6 are facing more risks. These two stations have higher concentrations of PM_2.5_ and heavy metals than other stations due to their proximity to the power plant, the carbon block factory, and high vehicle traffic^51–53^. Two stations IS1 and IS6 are located near industries in addition to vehicle traffic, which are the main sources of heavy metal emissions in urban areas^66^. Hence, it is crucial to implement mitigation measures aimed at diminishing the presence of such pollutants in these areas.

### Risk assessment

Table 3 summarizes the carcinogenic and non-carcinogenic risk of heavy metal measured in ambient air. As shown in Table 3, the LTCR value for all metals was as follows: Cr > Cd > Ni > As. The value of LTCR attributed to Cr in winter and summer as well as urban and industrial areas was higher than the background value (10^–4^). This study showed that the LTCR of chrome metal in the summer season in the urban area for infants is also higher than the background value. In addition, the highest LTCR value of Cr was found in industrial area and winter season For adults (1.97E−03). Also, according to the age groups, the highest risk of carcinogenesis was calculated for adults > children > infants respectively. Kawichai et al. Rayong, Thailand showed that the carcinogenic risk of chromium for adults and children was higher than 10^–4^, for infants between 10^–4^ and 10^–6^, and the carcinogenicity of other metals was within the safe range^48^. In addition to the earth's crust, human activities can also increase the concentration of Cr in breathing air. Processes such as wear and mechanical friction of vehicles and smoke from car exhausts can be factors of increasing Cr in the air of cities^67^. These results indicated that inhalation of Cr bound with PM_2.5_ can be carcinogenic for residents of Ahvaz city and especially those who live in the proximity of industrial areas. On the other hand, in case of other metals, the value of LTCR were within the recommended by USEPA (10^–4^ to 10^–6^), indicating a tolerable risk for the residents of Ahvaz. Moreover, the non-carcinogenic risk of the studied metals was as follows: Ni > Cd > As > Mn > Cr > Hg > Al. In this study, the HQ value for Cd, Ni and As in winter and summer and all three age groups were higher than one. In addition, the highest amount of HQ was obtained, respectively Cd (4.61), Ni (3.47) and As (1.88). These results show that inhalation of these metals can be a health risk for the residents of Ahvaz city. Wang, X., et al. (2018) reported that Pb for children and adults and arsenic metal for coconut had carcinogenic risk, and the total carcinogenic risk of metals for children and adults was 4.64 × 10^–4^ and 3.12 × 10^–4^ , respectively^68^. Li, et al. reported that the As (3.07), Mn (3.06) and Cd (1.2) have non-carcinogenic health effects above the safe limit (HQ = 1) and chromium metal for children 1.44. E 10^–4^ and adults 5.76E 10^–4^ was carcinogenic^69^. Sakunkoo et al. study was conducted in 2022 in four areas (academic, residential, industrial and agricultural) of Khon Kaen, Thailand to assess the risk of carcinogenicity of metals bonded with PM_2.5_. This study showed that industrial residents (especially children) have a higher risk of carcinogenesis than other areas due to the high concentration of PM_2.5_ and heavy metals^70^.

**Table 3:** Carcinogenic and non-carcinogenic risk of heavy metals in winter and summer in urban and industrial areas.

			Age group	Al	Ni	Cr	Cd	Mn	As	Hg
Winter	Industrial Min	HQ_inh_	< 2	3.75E−02	3.29028	1.99 E−01	3.71E+00	8.87 E−01	1.88E+00	6.07 E−02
			2–16	3.75 E−02	3.29028	1.99 E−01	3.71E+00	8.87 E−01	1.88E+00	6.07 E−02
			16–70	3.75 E−02	3.29028	1.99 E−01	3.71E+00	8.87 E−01	1.88E+00	6.07 E−02
		LTCR	< 2	*	3.16 E−07	4.78 E−05	1.91 E−06	*	1.91 E−06	*
			2–16	*	2.21 E−06	3.35 E−04	1.33 E−05	*	1.33 E−05	*
			16–70	*	8.69 E−06	1.32 E−03	5.24 E−05	*	5.24 E−05	*
	Urban	HQ_inh_	< 2	3.86 E−02	3.47443	1.62 E−01	3.76E+00	7.31 E−01	1.19E+00	4.13 E−02
			2–16	3.86 E−02	3.47443	1.62 E−01	3.76E+00	7.31 E−01	1.19E+00	4.13 E−02
			16–70	3.86 E−02	3.47443	1.62 E−01	3.76E+00	7.31 E−01	1.19E+00	4.13 E−02
		LTCR	< 2	*	3.34 E−07	3.88 E−05	1.94 E−06	*	1.53 E−07	*
			2–16	*	2.33 E−06	2.72 E−04	1.36 E−05	*	1.07 E−06	*
			16–70	*	9.17 E−06	1.07 E−03	5.32 E−05	*	4.21 E−06	*
Summer	Industrial Min	HQ_inh_	< 2	2.72 E−02	2.85218	1.66 E−01	4.61E+00	7.05 E−01	1.14E+00	5.08 E−02
			2–16	2.72 E−02	2.85218	1.66 E−01	4.61E+00	7.05 E−01	1.14E+00	5.08 E−02
			16–70	2.72 E−02	2.85218	1.66 E−01	4.61E+00	7 E−01	1.14E+00	5.08 E−02
		LTCR	< 2	*	2.74 E−07	3.99 E−05	2.37 E−06	*	1.46 E−07	*
			2–16	*	1.92 E−06	2.79 E−04	1.66 E−05	*	1.02 E−06	*
			16–70	*	7.53 E−06	1.10 E−03	6.52 E−05	*	4.02 E−06	*
	Urban	HQ_inh_	< 2	2.71 E−02	2.64190	1.62 E−01	4.18E+00	8.6 E−01	1.10E+00	4.16 E−02
			2–16	2.71 E−02	2.64190	1.62 E−01	4.18E+00	8.6 E−01	1.10E+00	4.16 E−02
			16–70	2.71 E−02	2.64190	1.62 E−01	4.18E+00	8.6 E−01	1.10E+00	4.16 E−02
		LTCR	< 2	*	2.54 E−07	3.89 E−05	2.15 E−06	*	1.41 E−07	*
			2–16	*	1.78 E−06	2.72 E−04	1.50 E−05	*	9.87 E−07	*
			16–70	*	6.97 E−06	1.07 E−03	5.91 E−05	*	3.88 E−06	*

## Conclusion

To the best of our knowledge, this study, for the first time investigate the concentration, spatial distribution, seasonal changes of PM_2.5_ and heavy metals, the carcinogenic and non-carcinogenic risk of heavy metals bounded PM_2.5_ and the survival rate of A549 human lung cells contacted to the PM_2.5_ in all areas of Ahvaz city as the mega city considering land use (urban and industrial). The results revealed that the level of PM_2.5_ and some heavy metals such as As, Ca, Cr and Mn were several times higher than the WHO guidelines. Considering that the concentration of PM2.5 and heavy metals was high in this study. Also because of the effects these pollutants can have on human health. It is expected that the health and environment trustees of Ahvaz city will consider appropriate management measures such as suitable fuel and increasing the quality of fuel consumed by vehicles and industries. The limitations could be considered for this study are: First, the concentration of heavy metals measured in this study was limited to PM_2.5_-bound values and other particles were not considered. Therefore, the concentration of heavy metals in the air of Ahvaz city can be higher than the measured values. Due to time and financial constraints, this study was conducted in Ahvaz city in two seasons, it is suggested to conduct similar studies in other cities of Khuzestan. Based on the findings of this study, it is necessary to implement management strategies and preventive measures to effectively regulate the levels of these pollutants in the city of Ahvaz.

## Data Availability

The data supporting the findings of this article is included within the article.
